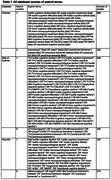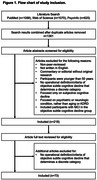# Objective Criteria for Subtle Cognitive Decline in Aging and Preclinical AD: A Systematic Review

**DOI:** 10.1002/alz.089283

**Published:** 2025-01-03

**Authors:** Kelsey R. Thomas, Emily C. Edmonds

**Affiliations:** ^1^ VA San Diego Healthcare System, San Diego, CA USA; ^2^ University of California, San Diego, La Jolla, CA USA; ^3^ Banner Alzheimer’s Institute, Tucson, AZ USA

## Abstract

**Background:**

The field of Alzheimer’s disease and related dementias (ADRD) has continued to move toward earlier detection. Within the 2018 NIA‐AA AD Research Framework, clinical stage 2 recognizes that someone can be cognitively unimpaired but still experience “subtle cognitive decline” as measured by subjective report or evidence of objective decline using neuropsychological measures. While significant attention has been given to methods of assessing subjective cognitive decline, there are no systematic examinations of the operational definitions of subtle cognitive decline using objective neuropsychological measures.

**Method:**

We performed a systematic literature search in PubMed/MEDLINE, Web of Science, and PsycInfo databases for articles with dates ranging from the start of the database through 11/1/2023. Search terms are included in Table 1. Only research studies that included participants aged 50+ without MCI/dementia and described a discrete objective subtle cognitive decline category were included.

**Result:**

Of 1361 publications that were initially identified, 73 met criteria for inclusion after a full review of the article (Figure 1). Results showed multiple methods for defining subtle cognitive decline. The 6 most common were: 1) subtle cognitive decline based on a specified cut‐off on a single cognitive test; 2) objectively‐defined subtle cognitive decline (Obj‐SCD) using cut‐offs (e.g., ‐1 SD) on multiple individual neuropsychological measures; 3) cut‐off (e.g., 10^th^ percentile) on a cognitive composite score; 4) “Pre‐MCI” criteria defined using a CDR of 0.5 but normal performance on neuropsychological testing; 5) cut‐off based on longitudinal rate of cognitive decline (e.g., over 1 year); and 6) data‐driven/clustering approach to classification. Each method identified a group that had worse AD‐related outcomes relative to cognitively normal participants.

**Conclusion:**

Across multiple methods, there is consistent evidence that objective subtle cognitive decline can be detected prior to MCI and is associated with ADRD biomarkers, neuroimaging results, and faster rates of progression to MCI/dementia. Notably, there is a lack of research of these subtle cognitive decline definitions in racially/ethnically diverse older adults, which is a significant weakness in the literature. Results of our review have implications for clinical trial recruitment and for the ethics and potential use of objective subtle cognitive decline criteria in clinical practice in the future.